# Magnetic Gel Composites for Hyperthermia Cancer Therapy

**DOI:** 10.3390/gels1020135

**Published:** 2015-09-30

**Authors:** Marleen Häring, Jana Schiller, Judith Mayr, Santiago Grijalvo, Ramon Eritja, David Díaz Díaz

**Affiliations:** 1 Institut für Organische Chemie, Universität Regensburg, Universitätsstr. 31, Regensburg 93040, Germany; E-Mails: Marleen.Haering@chemie.uni-regensburg.de (M.H.); Jana.Schiller@chemie.uni-regensburg.de (J.S.); Judith.Mayr@chemie.uni-regensburg.de (J.M.); 2 IQAC-CSIC, Jordi Girona 18-26, Barcelona 08034, Spain; E-Mails: sgrgma@cid.csic.es (S.G.); recgma@cid.csic.es (R.E.); 3 The Biomedical Research Networking Center in Bioengineering, Biomaterials and Nanomedicine (CIBER-BBN), Jordi Girona 18-26, Barcelona 08034, Spain

**Keywords:** hydrogel, composites, magnetic nanoparticles, hyperthermia, cancer therapy, drug delivery

## Abstract

Hyperthermia therapy is a medical treatment based on the exposition of body tissue to slightly higher temperatures than physiological (*i.e.*, between 41 and 46 °C) to damage and kill cancer cells or to make them more susceptible to the effects of radiation and anti-cancer drugs. Among several methods suitable for heating tumor areas, magnetic hyperthermia involves the introduction of magnetic micro/nanoparticles into the tumor tissue, followed by the application of an external magnetic field at fixed frequency and amplitude. A very interesting approach for magnetic hyperthermia is the use of biocompatible thermo-responsive magnetic gels made by the incorporation of the magnetic particles into cross-linked polymer gels. Mainly because of the hysteresis loss from the magnetic particles subjected to a magnetic field, the temperature of the system goes up and, once the temperature crosses the lower critical solution temperature, thermo-responsive gels undergo large volume changes and may deliver anti-cancer drug molecules that have been previously entrapped in their networks. This tutorial review describes the main properties and formulations of magnetic gel composites conceived for magnetic hyperthermia therapy.

## 1. Introduction

The application of heat to treat certain medical conditions, including possible tumors, has a long history. Ancient Greeks, Romans, Indians, and Egyptians used heat to treat breast masses, which is still a recommended self-care treatment for breast engorgement [1]. Nowadays, hyperthermia therapy [2] constitutes a medical treatment based on the exposition of body tissue to slightly higher temperatures than physiological (*i.e.*, between 41–46 °C) to damage and kill cancer cells or to make them more sensitive to the effects of radiation and anti-cancer drugs [3,4]. Despite still being an experimental technique, local hyperthermia has shown in clinical trials to be effective when combined with well-developed chemotherapy or radiotherapy for cancers such as breast, cervical, prostate, head and neck, melanoma, soft-tissue sarcoma, and rectal cancer limited to small areas, among others [5,6].

Hyperthermia alters the cell walls by means of so-called heat shock proteins and increases blood flow to the warmed area that can enhance the delivery of drugs [3]. Hyperthermia also increases oxygen (a potent radiosensitizer) delivery to the area making the tumor cells overacidified, which leads to a lack of nutrients in the tumor. This, in turn, disrupts the metabolism of the cells so that cell death (apoptosis) can set in. Higher oxygen concentrations can also make radiation more likely to damage and kill cells by forming DNA-damaging free radicals [7], as well as preventing cells from repairing the damage induced during the radiation session [8]. It should be emphasized that cancerous cells are not inherently more susceptible to the effects of heat than normal cells [3]. However, the vascular disorganization of a solid tumor results in an unfavorable microenvironment inside tumors. Consequently, the tumor cells are already stressed by low oxygen, higher than standard acid concentrations and insufficient nutrients, being significantly less able to tolerate the added stress of heat than a healthy cell in normal tissue.

There are many methods by which heat may be delivered to tumor areas, including ultrasound, microwave, induction heating, infrared radiation, radiofrequency, and magnetic hyperthermia. The latter refers to the introduction of magnetic particles [9] into the tumor tissue, followed by the application of an external alternating magnetic field (AMF) [10,11]. The particles transform the energy of the magnetic field into heat by several mechanisms: eddy current loss (minor effect in ferromagnetic particles, such as Fe_3_O_4_, due to their low electrical conductivity), hysteresis loss during reversal magnetization (a major contribution in most cases), and relaxation loss, including Brownian relaxation and Neel relaxation (main contributions in superparamagnetic materials due to zero remanence). The efficiency of energy transformation is strongly dependent on the strength and frequency of the applied magnetic field, the properties of the magnetic particles (e.g., size, distribution), and the cooling capacity of the surrounding flow [12,13].

**Figure 1:**
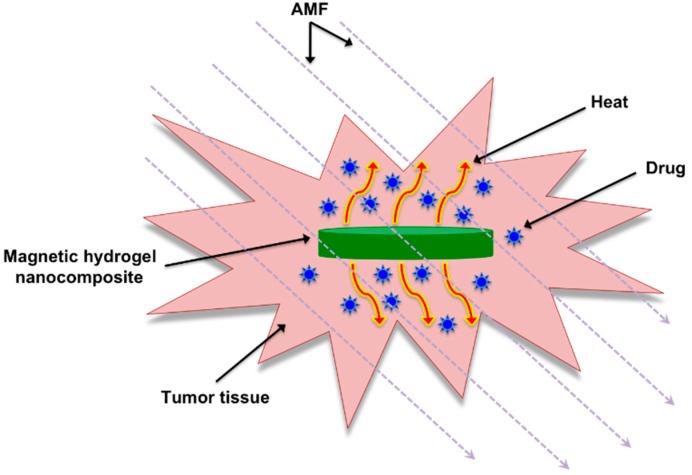
Basic illustration of thermo-responsive magnetic gel composites that can be heated upon exposure to an AMF allowing for the controlled delivery of entrapped drugs.

An interesting approach for magnetic hyperthermia is the use of biocompatible magnetic gel composites made by incorporation of magnetic micro- and/or nanoparticles into polymer hydrogel matrices [14,15,16,17,18,19]. Magnetite (Fe_3_O_4_) provides the most attractive magnetic material of common use due to its strong magnetic property and low toxicity [20]. On the other hand, cross-linked polymeric hydrogels have properties that make them suitable for a wide range of biomedical applications due to their resemblance to natural living tissue and inherent biocompatibility [21,22,23,24], which can be partially attributed to their soft, flexible nature and high water content [25,26]. One of their most useful properties is the ability to undergo abrupt changes in volume without dissolving in the immersed medium. So-called “smart gels” are able to swell or shrink up to 1000 times in response to small changes in temperature, pH, electric fields or solvent and ionic composition [27]. Due to the hysteresis loss from the magnetic particles, the temperature of the system increases and it crosses the lower critical solution temperature (LCST), thermo-responsive gels undergo large contraction and may deliver drug molecules entrapped in their networks in a controlled manner [26,28] (Figure 1).

**Figure 2:**
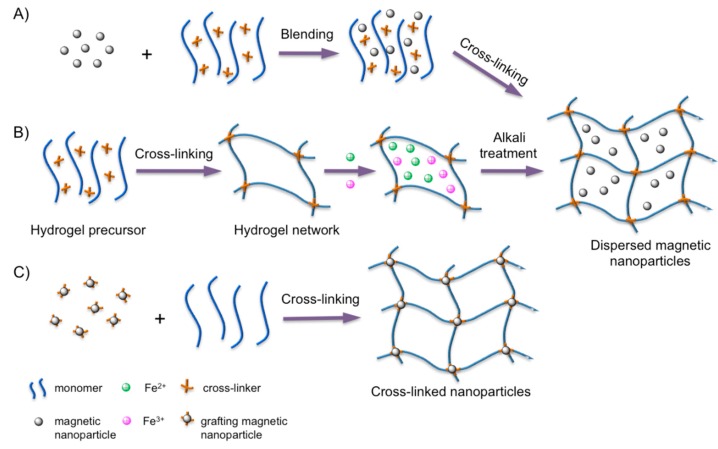
Main preparation methods of magnetic hydrogels. (**A**) The blending method: the magnetic nanoparticles are mixed with a hydrogel precursor solution at a certain molar ratio and cross-linked; (**B**) the *in situ* preparation method: the magnetic nanoparticles are fabricated via an *in situ* precipitation in the network of the polymer hydrogel after the cross-linking; and (**C**) the grafting-onto method: grafting several functional groups onto the surface of the magnetic nanoparticles to be used as cross-linkers. Adapted with permission from reference [32]. Copyright ^©^ 2013 Wiley-VCH.

Figure 2 shows the three main methods to prepare magnetic hydrogels: the blending method [29], the *in situ* synthesis [30], and the grafting-onto approach [31]. Variables, such as the type of gel network and magnetic nanoparticles, as well as their concentrations, should be considered when preparing these composites for biomedical applications [32] including magnetic hyperthermia cancer therapy [33].

This tutorial review focuses on the properties and formulations of magnetic gel composites conceived mainly for magnetic hyperthermia therapy mainly in combination with drug delivery. Non-magnetic gels [34] or magnetic composites based on non-gel formulations [35] for magnetic hyperthermia and magnetic gels for other applications [36] are out of the scope of this manuscript, although some of these materials are referenced for the purpose of better understanding.

## 2. Development of Magnetic Gel Composites for Hyperthermia Therapy

### 2.1. Magnetic Gel Composites Based on Natural Polymers

More than two decades ago, Jordan and co-workers [37] reported the study of subdomain ferrite particle suspensions (SDP, stabilized dextran-coated particles with Ø ≈ 3–10 nm) and multidomain ferrite particle suspensions (MDP, few micrometers or more in diameter) embedded in a 2% agar hydrogel matrix and exposed to an AMF at exact frequency and magnetic field strength (*H*). The agar hydrogel without ferrite material served as control and the experimental results indicated a superior performance of SDP with respect to their specific absorption rate (SAR = amount of heat released by a unit weight of material per unit time). The authors also developed a solid-state physical model to explain the specific properties of magnetic fluids with respect to a possible use in hyperthermia. After this work, Andrä and co-workers [38] also developed a predictive mathematical model to calculate the spatial temperature distribution of a small spherical region (Ø = 6.3 mm) containing magnetic particles (Fe_3_O_4_) embedded in a carrageenan gel as a function of the time exposed to an AMF at a frequency of 400 kHz and 6.5 kA/m of amplitude (SAR = 365 W/g).

**Figure 3:**
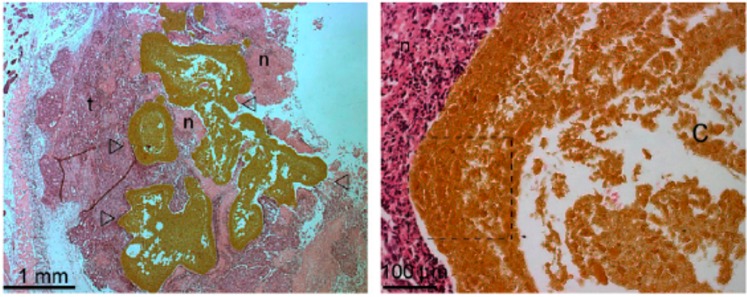
Photomicrographs (right: 25×, left: 200×) of Swiss nude mice subcutaneous xenografts Co112 injected with an EVAL-based formulation incorporating 40% *w*/*v* of magnetic nanoparticles. Empty arrow heads (Δ) indicate the implant; t, the tumor tissue; and n, the areas of necrosis. On the left, the central distribution of the implant in the tumor with extensions toward its periphery. This high microparticles load masks some characteristics of type III microstructure in the large peripheral rim concentrating microparticles (dashed lined box), where implants devoid of microparticles show a remarkably thin initially precipitated skin layer and a porous sub-layer. However, lacunas observed at low magnification in the center of implant (C) support type III microstructure. Adapted with permission from reference [42]. Copyright ^©^ 2010 Elsevier.

Injectability is another important property to consider for *in vivo* applications of magnetic hydrogels in cancer therapy to target diseased sites with minimal invasiveness [39]. A number of hydrogels are known to undergo solution-to-gelation phase transition after injection through chemical or physical cross-linking including thermal-, pH- or ion-induced [40,41]. Within this context, Jordan and co-workers [42] investigated the use of several stimuli-responsive polymers for cancer therapy, including thermo-sensitive (e.g., chitosan, poloxamer 407) and ion-responsive polymers (e.g., sodium alginate), embedded with superparamagnetic silica iron oxide nanoparticles (SPIONs). Magnetic hydrogel, single-solvent organogel and cosolvent (low-toxicity hydrophilic solvent) organogel formulations were injected into human cancer tumors xenografted in mice. The thermo-responsive chitosan and poloxamer-based hydrogels, which accommodated 20% *w*/*v* of the magnetic particles, proved to be deficient for *in situ* implant formation at higher temperature caused by an AMF. On the other hand, alginate hydrogels incorporated 10% *w*/*v* of SPIONs and the external ion-induced gelation led to strong implants localizing to the tumor periphery. However, the internal gelation failed *in situ*. The organogel formulations, which consisted of precipitating different polymers (e.g., poly(ethylene-*co*-vinyl alcohol) EVAL, polyurethane, cellulose) dissolved in single organic solvents, displayed various microstructures. Specifically, a 8% EVAL in DMSO containing 40% *w*/*v* of magnetic particles formed the most suitable implants in terms of tumor casting and heat delivery (Figure 3). However, formulations with 20% *w*/*v* magnetic particles were desired due to reduced toxicity and centered tumor implantation. Moreover, generation of sufficient heat by increasing the content of magnetic particles, enhancing the hydrogel viscosity after intratumoral injection to avoid undesired migration of the particles and their elimination from the body afterwards constitutes the main aspects to consider for further optimization of these systems. Hoare and co-workers [43] have recently demonstrated that nanocomposite *in situ*-gelling hydrogels containing both SPIONs and thermoresponsive microgels facilitate pulsatile, high-low release of a model drug via an AMF.

The accumulation of magnetic nanoparticles in the tumor is an essential requirement to carry out efficient magnetic hyperthermia treatments. However, it has been described that intratumoral injection may induce heterogeneous distribution of the injected materials [44,45]. Although this heterogeneous nanoparticle distribution could reduce the therapeutic effect and it is difficult to circumvent, there are some promising examples in order to minimize them. Thus, Dutz and co-workers [46] described that around 85% of administered nanoparticle were immobilized to tumor tissue when using gelatin. Although several examples can be found in the literature regarding the use of hydrogels *in vivo* for cancer treatment [47,48], the homogeneity in the injection site should be carefully studied.

**Figure 4:**
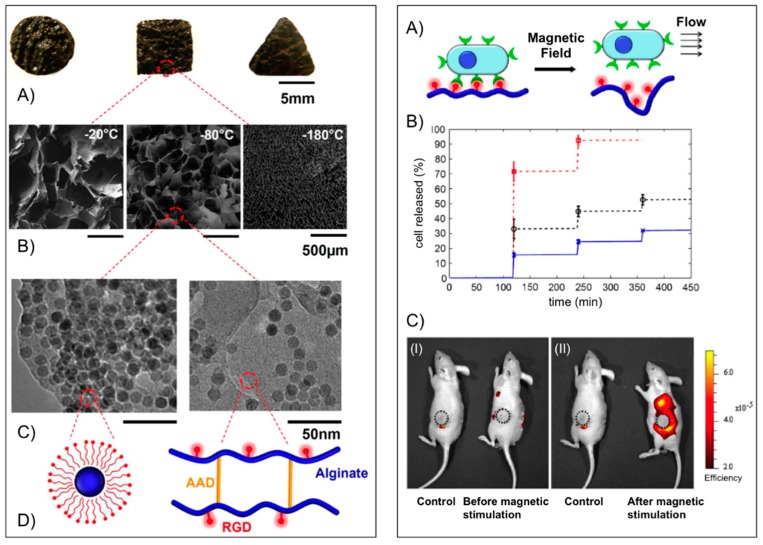
*Left panel*: (**A**) Photograph of bulk gels with various shapes; (**B**) SEM images of scaffold with various pore size (average diameter from left to right: 700, 300 and 20 μm) and pore connectivity in the ferrogels; (**C**) TEM images of iron oxide nanoparticles in the gel at different concentrations (*left*: 13 wt %; *right*: 4 wt %); and (**D**) schematic plots of the nanoparticles coated with Pluronic F127 (*left*) and alginate covalently cross-linked by AAD and coupled with RGD peptides (*right*). *Right panel*: (**A**) schematic plot of gel deformation and resulting water convection inducing cell release from macroporous gels; (**B**) cumulative release profiles of fibroblasts from macroporous ferrogels with 100% (cross), 50% (circle) and 10% (square) of the baseline RGD density, following application of cycled magnetic field; and (**C**) *in vivo* fluorescence images of mice implanted with macroporous ferrogel containing mouse mesenchymal stem cells stained with DiOC18 before (I) and after (II) magnetic stimulation. The control case was subjected to no magnetic stimulation. The positions of the gel discs are indicated by circles on the figure. Adapted with permission from reference [49]. Copyright ^©^ 2011 National Academy of Sciences.

Mooney’s group [49] used alginate to fabricate macroporous ferrogels capable of on-demand drug and cell delivery under the control of a moderate magnetic field due to a large deformation and volume change (over 70%) of the hydrogel. For potential *in vivo* applications peptides containing the arginine-glycine-aspartic acid (RGD) amino acid sequence were first covalently coupled via carbodiimide chemistry to the alginate yielding a conjugate with 7.43 μmol RGD per gram of alginate. The RGD coupling confers a specific mechanism for integrin-mediated cell adhesion to the otherwise non-adhesive polymer. Fe_3_O_4_ nanoparticles (Ø ≈ 10 nm), precoated with Pluronic F127 to minimize agglomeration, were then embedded into the functionalized alginate in the presence of adipic acid dihydrazide (AAD), which acted as cross-linker to maintain the macroporous structure following lyophilization and subsequent rehydration (Figure 4, *left panel*). The porous hydrogel scaffold could act as a depot and facilitate the *in vitro* controlled release of different active substances including mitoxantrone, an antineoplastic agent, a plasmid DNA (*M_r_ ~* 10^6^) condensed with polyethyleneimine and the chemokine SDF-1α (*M_r_ ~* 8000) from the scaffold. In each case, the cumulative release profile showed a stepwise increment with magnetic stimulation. The capability of the macroporous ferrogels in cells delivery under magnetic stimulation *in vivo* was also demonstrated using mouse mesenchymal stem cells stained with DiOC18, a membrane dye with near infrared emission maximum. One hour after the implantation, the gels were subjected to 120 cycles (on/off) of the external magnetic field by approaching and retracting a magnet against the mouse skin over the gel. The control mouse (no magnetic stimulation) showed practically no change in fluorescence, whereas application of the external magnetic field led to a significant increase in fluorescence around the ferrogel, indicating a burst release of stem cells (Figure 4, *right panel*). The peptide-modified macroporous ferrogel also maintains the adhesion and viability of the resident cells, and subjecting the gels to external magnetic stimulation allows one to release a prescribed number of the resident cells on demand over various time frames. Multiple parameters are tunable in this system, including peptide density, the strength of applied magnetic field, number of magnetic cycles, and frequency of magnetic stimulation, to control the release of various cell types on-demand.

**Figure 5:**
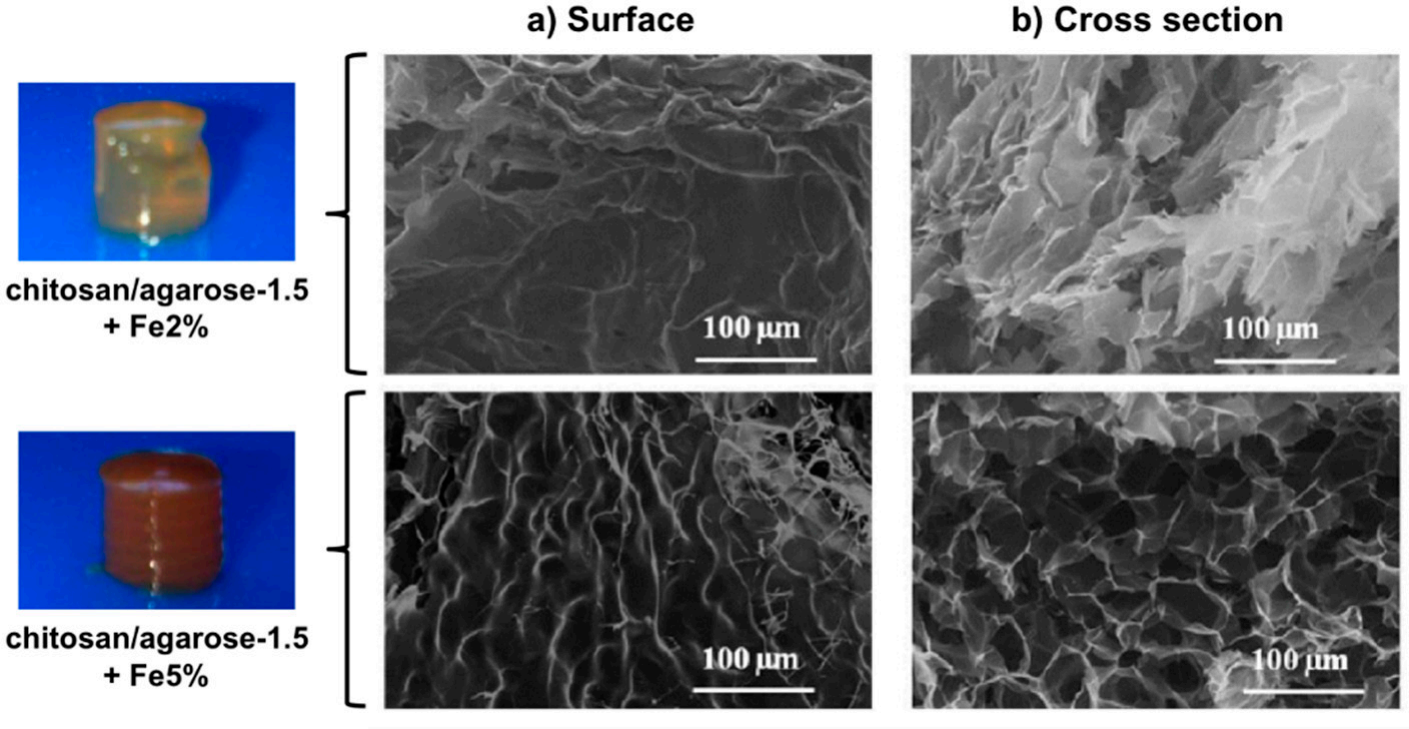
Photographs of chi/aga-1.5 ferrogels loaded with magnetic nanoparticles at 2.0% and 5.0% (*w*/*v*) and their SEM images corresponding to the (**a**) surface and (**b**) cross section. Adapted with permission from reference [51]. Copyright ^©^ 2015 MDPI.

Hernández, Mijangos and co-workers [50] reported a series of ferrogels derived from Fe_3_O_4_ nanoparticles (synthesized *in situ*, Ø ~ 10 nm) embedded in alginate or chitosan combined with thermally responsive poly(*N*-isopropylacrylamide) (PNIPAm). SAR values between 100 and 300 W/g were obtained upon application of an AMF (260 kHz, 16 mT), which was enough for attaining the LCST of the polymeric matrix within few minutes, making them good candidates for magnetic hyperthermia. Very recently, the same research team [51] reported the preparation of microporous ferrogels by encapsulating the magnetic nanoparticles at two different concentrations (2.0% and 5.0% *w*/*v*) within mixed chitosan/agarose (chi/aga) hydrogels having different concentrations of agarose (1.0%, 1.5%, and 2.0% *w*/*v*) and a fixed concentration of chitosan (0.5% *w*/*v*) (Figure 5). Thermogravimetric measurements showed that ferrogels present higher degradation temperatures than the control chitosan/agarose hydrogels without magnetic nanoparticles, suggesting possible interactions between the magnetic nanoparticles and the polymer composite matrix. This also prevented to some extent agarose gelation. Moreover, the ferrogels were also able to heat in response to the application of an AMF (418.5 kHz and 24 kA/m). The observed increase of temperature with time depended on the iron concentration (*i.e.*, Δ*T* ~ 2.5 °C for Chi/Aga-1.5 + Fe5%; Δ*T* ~ 1.5 °C for Chi/Aga-1.5 + Fe2%), although SAR values remained almost constant (Table 1). A significant decrease of SAR with respect to the ferrofluid was ascribed to possible differences in the agglomeration of the magnetic material in the ferrogels.

**Table 1:** Specific power absorption of the samples reported in reference [51].

Samples	Δ*T*/Δ*t* (°C/s)	Water Content (%)	Fe_3_O_4_ (mg/mL)	SAR (W/g)
Ferrofluid	1.61	89.7	69.9	96
Chi/Aga-1.5 + Fe2%	0.007	96.8	0.96	30
Chi/Aga-1.5 + Fe5%	0.009	97.1	1.16	32

**Figure 6:**
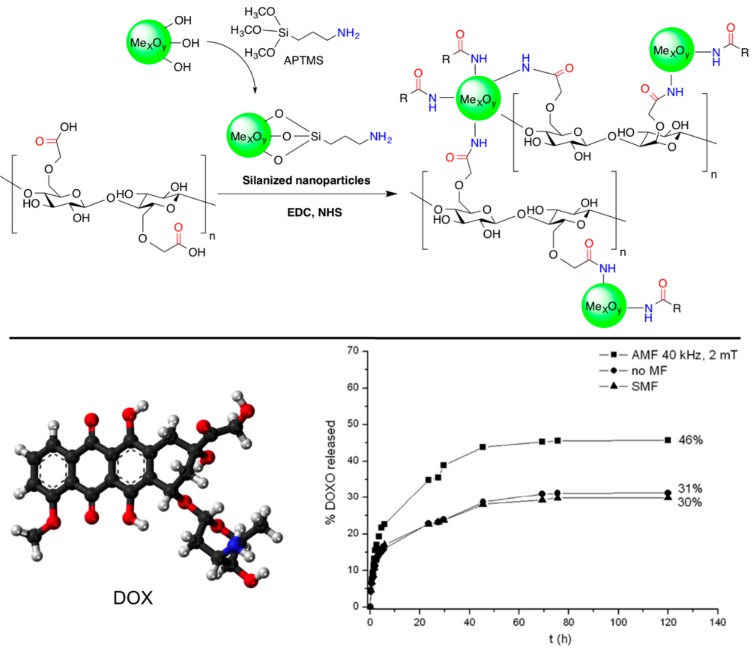
*Top*: Reaction scheme of the formation of CMC hybrid hydrogels. The reaction involves the formation of an amide bond between the carboxylic groups of CMC and the amine groups of the amino-functionalized metal oxide nanoparticles (cross-linkers) in the presence of EDC and NHS; *Bottom*: Ball-and-stick molecular model of DOX (doxorubicin) and comparison of its release from Fe_3_O_4_-CMC hydrogel composite in NaCl 0.15 M in the absence of magnetic field (circles), in the presence of an AMF (squares) and with SMF (triangles). Adapted with permission from reference [52] and reference [54]. Copyrights ^©^ 2011 Royal Society of Chemistry and ^©^ 2015 MDPI, respectively.

Barbucci and co-workers [52,53] reported the preparation of new non-toxic hybrid hydrogels based on the covalent binding of magnetic CoFe_2_O_4_ magnetic nanoparticles to carboxymethylcellulose (CMC). The nanoparticles were first functionalized with (3-aminopropyl)-trimethoxysilane (APTMS) in order to introduce amino groups on the surface (the magnetic properties of the nanoparticles are not influenced significantly by the silanization treatment [54]). Subsequent EDC coupling with the carboxylic groups of CMC yielded the corresponding hybrid biomaterial (Figure 6, *top*). The authors incorporated methylene blue (MB) into the composite as a model drug to confirm its controlled release when exposed to an AMF, especially at low frequency (4 Hz) and high magnetic intensity (0.5 T) [53]. Very interestingly, no heating effect on the sample was apparently observed in this case, suggesting the possibility of a different release mechanism. However, hyperthermic properties were observed at higher frequency and field amplitude [54]. Additional experiments using composites with different amounts of magnetic nanoparticles (*i.e.*, 50% and 70% with respect to the quantity of the polymer) showed different responses to the applied magnetic field [55]. The greater number of nanoparticle hydrogel led to the formation of some nanoparticle clusters limiting the drug release; conversely, lower amounts of nanoparticles showed a higher release of MB over time. Moreover, it was found that the release behavior of these hybrids could be modulated by applying alternating and static magnetic fields in a cyclic manner. The application of an AMF induced a higher release of MB than in the absence of a magnetic field. In contrast, when the hybrid hydrogels were exposed to static magnetic fields (SMF) the release of MB was slowed down. This was correlated with the water uptake (WU) (*i.e.*, WU (AMF) > WU (no MF) > WU (SMF)), and suggested a possible explanation for the release mechanism based on structural modifications of the polymeric chains that occurs when the hybrid hydrogels are exposed to the magnetic fields. Thus, SMF seemed to induce a lengthening and thinning of the material, with a consequent reduction in the pore sizes, whereas the AMF driven the formation of more open structures [55].

The same synthetic strategy was also applied to combinations with other biopolymers and nanoparticles, such as hyaluronic acid (HYAL) and Fe_3_O_4_, respectively [56]. The hybrid hydrogels were previously loaded with anticancer doxorubicin (DOX) and its release was induced by exposition of the composite to an AMF (Figure 6, *bottom*). In the case of HYAL, the release of DOX was found to be much greater than that from the analogous hybrid based on CMC under the same magnetic field. This was correlated to the rheological properties of the gels, which showed a lower elastic modulus of the HYAL-based composite, making the release of the drug more favorable.

**Figure 7:**
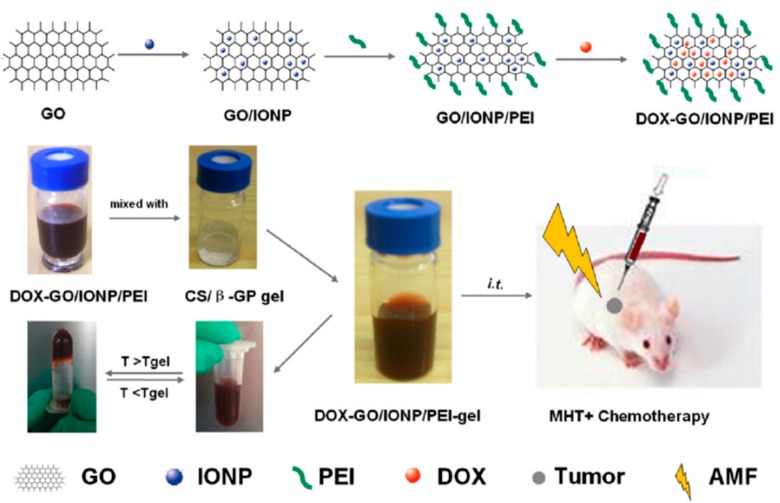
Schematic illustration of DOX-GO/IONP/PEI-gel. MHT = Magnetic hyperthermia. Reprinted with permission from reference [57].

**Figure 8:**
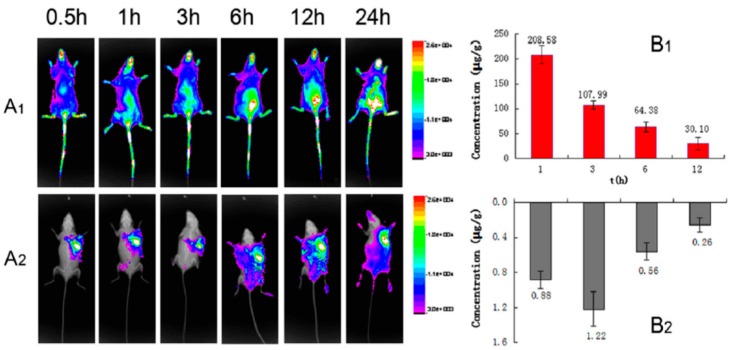
(**A**) Time-dependent biodistribution assay *in vivo* of S180 tumor-bearing mice. (**A**) FX imaging *in vivo*; ((**A_1_**) IR 783, i.v.; (**A_2_**) DOXGO/IONP/PEI-gel, intratumorally injected); (**B**) the drug distribution of (**B_1_**) DOX (free IR783) ,and (**B_2_**) DOX-GO/IONP/PEI-gel (intratumorally injected) in tumor tissues (*n* = 6). Reprinted with permission from reference [57].

Zhang’s group [57] has described a novel locally injectable, biodegradable, and thermo-sensitive hydrogel made from chitosan and β-glycerophosphate. It incorporated polyethylenimine (PEI)-modified super-paramagnetic graphene oxide (GO/IONP/PEI) as a form of minimally-invasive treatment of cancer lesions by magnetically-induced local hyperthermia. DOX was mixed into the hydrogel which was pre-loaded on GO/IONP/PEI to create a drug delivery system DOX-GO/IONP/PEI-gel (Figure 7). In addition to the physicochemical properties, magnetic properties, and DOX release profile of the DOX-GO/IONP/PEI-gel, the authors also evaluated the materials both *in vitro* and *in vivo*. The aqueous solution of the hydrogel showed a sol-gel transition behavior depending on temperature changes. Magnetization loops indicated the super-paramagnetic properties of GO/IONP/PEI. This composite did not show obvious toxicity to MCF-7 cells alter 72 h incubation, having almost no influence on the cellular cycle. Compared with free DOX, DOX-GO/IONP/PEI could efficiently pass through cell membranes, leading to more apoptosis and demonstrating higher antitumor efficacy on MCF-7 cells *in vitro*. Furthermore, DOX-GO/IONP/PEI-gel intratumorally injected showed high antitumor efficacy on tumor-bearing mice *in vivo* (Figure 8). The antitumor efficacy via cell apoptosis was higher when combined with an AMF (488 kHz, 20 A) for 20 min. Biodistribution was established based on real-time images of near-infrared fluorescent dye, IR783-labeled GO/IONP/PEI-gel and the free IR783 as the control in the tumor-bearing mice. After IR783-labeled GO/IONP/PEI-gel was intratumorally injected, considerable fluorescence signals were detected mainly in the tumor tissues of the mice. On the contrary, for the free IR783, the fluorescence signals were detected in the whole body of the mice and decreased more quickly.

### 2.2. Magnetic Gel Composites Based on Synthetic Polymers

**Figure 9:**
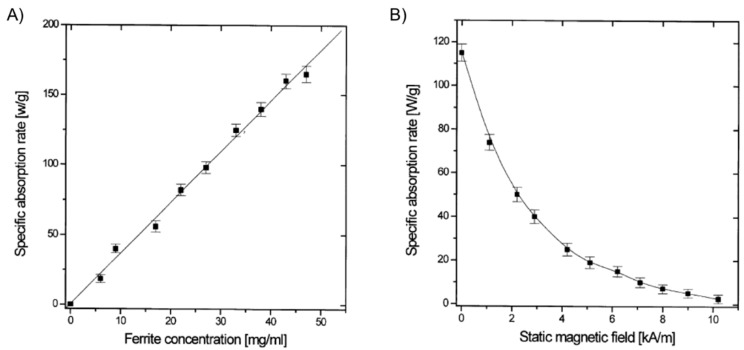
(**A**) SAR of gel samples with varying total ferrite concentrations in an AC of 217 kHz and 9.6 kA/m; and (**B**) dependence of SAR of a gel sample (ferrite concentration 30 g/L) on the SMF applied in the direction perpendicular to an AC field of 217 kHz and 9.6 kA/m. Adapted with permission from reference [14]. Copyright ^©^ 2001 Elsevier.

It was not until the beginning of the 21st century that the field expanded with the preparation of magnetic gels based on synthetic polymers that could be suitable for hyperthermia therapy. Babincová and co-workers [14] reported the study of superparamagnetic ferrite nanocrystals of *ca*. 10 nm within a gel network formed by bridging anionic bis(ethylhexyl) sodium sulfosuccinate (AOT) reverse micelles. Micelles of AOT were obtained by mixing FeSO_4_ solution (ca. 1 M) and AOT solution (0.5 M in isooctane) in a 1:11 volume ratio. Similarly, AOT micellar solution with NH_4_OH (volume ratio 1:8) was prepared and mixed with the previous iron-containing solution. Vigorous stirring for 2 h, solvent evaporation at 45 °C for 15 h, addition of isooctane to the obtained dry residue and 2,6-dihydroxynaphtalene (2,6-DHN) (molar ratio AOT/2,6-DHN = 60) gave a brown organogel formed through the hydrogen bonding of AOT sulfosuccinate head group and the hydroxyl groups on 2,6-DHN. In this strategy, the AOT reverse micelles were used as nanoreactors for the synthesis of ferrite nanocrystals. Moreover, the use of isooctane as solvent imparted excellent thermal stability to the organogels. The study of the heating properties of these magnetic gels in an AMF at a frequency of 217 kHz and 9.6 kA/m of amplitude revealed SAR values up to 150 W/g for ferrite concentrations up to 50 g/L (Figure 9). SAR was substantially reduced when the applied magnetic field strength (*H*) reached the value of the AC field. As expected, control experiments using gels prepared in the absence of ferrite particles showed no heating effect.

In 2004, Lao and Ramanujan [10] used micron-sized (*ca.* 3–5 μm) Fe_3_O_4_ particles dispersed in a polyvinyl alcohol (PVA) hydrogel through a conventional freezing/thawing method for hyperthermia applications. The mild magnetic properties and well-established biocompatibility and history of clinical usage of both Fe_3_O_4_ and PVA made them ideal candidates for the proof-of-concept with synthetic polymers. Systematic studies of input variables demonstrated a positive effect of both iron concentration and *H* on the maximum temperature that the system could reach as well as the rate of temperature increase (Δ*T*/Δ*t*) (*i.e.*, the sample was heated faster by the magnetic field). After reaching the maximum value the temperature then stabilized for a long time, indicating a good control of temperature and heat flux, a requirement for hyperthermia application. On the other hand, the SAR was found to increase with *H* but resulted practically insensitive to Fe_3_O_4_ content. The results showed that the Fe_3_O_4_-PVA ferrogel composite could steadily reach a maximum temperature of 47 °C at 2.0 wt % (Fe_3_O_4_ concentration) within *ca.* 6 min under an AMF with an amplitude of 2.0 kA/m and a frequency of 357 kHz (SAR = 5.8 W/g). The use of higher iron concentration (2.5 wt %) and lower magnetic field (1.7 kA/m) allowed reducing the maximum temperature to 43 °C (SAR = 3.8 W/g).

**Figure 10:**
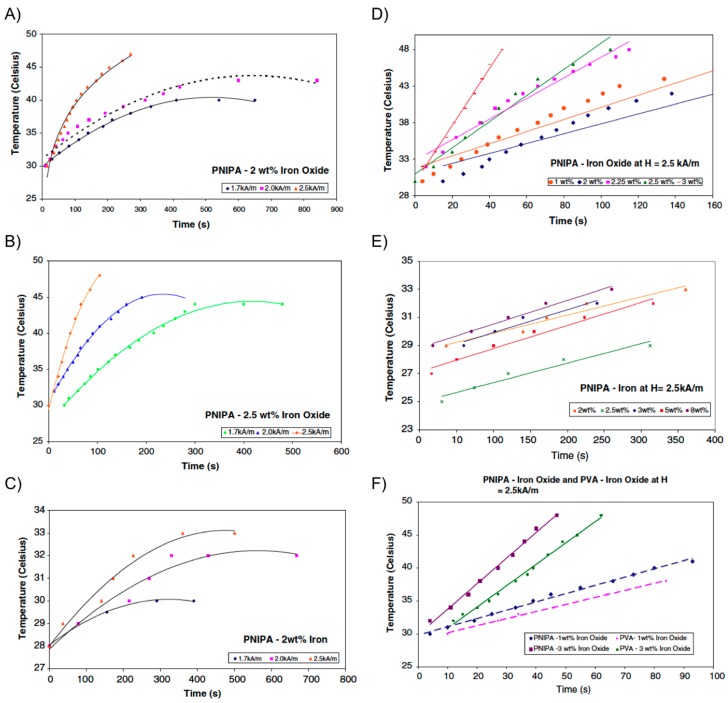
(**A**) Temperature *vs.* time for PNIPA-2 wt % iron oxide; (**B**) temperature *vs.* time for PNIPA-2.5 wt % iron oxide; (**C**) temperature *vs.* time for PNIPA-2 wt % iron; (**D**) temperature *vs.* time for PNIPA-iron oxide at *H* = 2.5 kA/m; (**E**) temperature *vs.* time for PNIPA-iron at *H* = 2.5 kA/m; and (**F**) temperature *vs.* time for PNIPA-iron oxide and PVA-iron oxide at *H* = 2.5 kA/m. Adapted with permission from reference [59]. Copyright ^©^ 2009 Springer.

A few years later, the same group [58,59] extended their work to evaluate the possibility of combining hyperthermia with drug delivery applications. The strategy involved the introduction of micron-sized iron particles (*i.e.*, iron oxide or iron powder) within PNIPAm hydrogel, rather than PVA, allowing for a collapse transition of the gel upon raising the temperature as the magnetic particles were heated. As described in the previous section, if drug molecules are dissolved in the hydrogel, they could be released during the volume phase transition across the LCST. Encapsulation of a dye into the composite served as a preliminary and qualitative demonstration of this application in these systems. The collapse transition temperature of both Fe_3_O_4_-PNIPAm and Fe-PNIPAm-composite hydrogels was 34 °C. After this temperature, the hydrogels changed from black to white in color and shrank in volume. Interestingly, the magnetic particles did not affect the collapse transition of the polymer network in the hydrogels. Same dependence of both the maximum temperature reached by the composite and the SAR with *H* and magnetic particles concentration, previously observed with Fe_3_O_4_-PVA hydrogels, was also observed for the PNIPAm-based composites (Figure 10). However, it was found that Fe_3_O_4_-PNIPAm hydrogel could be used for hyperthermia therapy but not Fe-PNIPAm because the latter was unable to reach the temperatures required for hyperthermia to be effective. Similar to Fe_3_O_4_-PVA, an optimum combination of particle concentration (2.5 wt % Fe_3_O_4_) allowed a maximum temperature of 45 °C within *ca*. 4 min under a field amplitude of 1.7 kA/m and a frequency of 275 kHz (SAR = 1.83 W/g).

**Figure 11:**
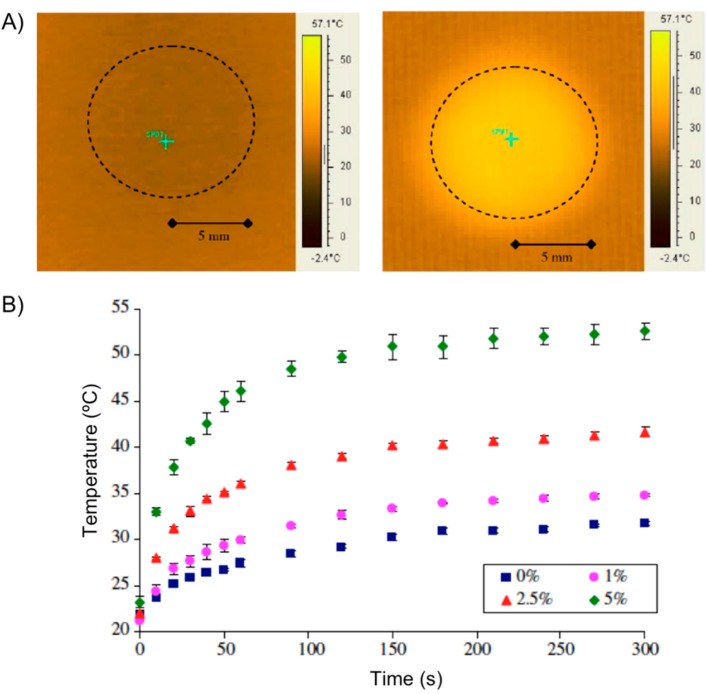
(**A**) Heating effect of nanocomposites in electromagnetic field: IR image of 5 wt % particle disc at 0 s (*left*) and at 60 s (*right*). The dotted circle shows the disc area; and (**B**) temperature increase of nanocomposites with varying particle loadings subjected to an electromagnetic field. % represents the particle loading by weight in the NIPAAm-PEG400DMA nanocomposite. Adapted with permission from reference [61]. Copyright ^©^ 2008 Elsevier.

During the NSTI-Nanotech 2008 conference, Ghosh and co-workers [60] reported the investigation of a similar hydrogel actuator using Fe_3_O_4_ particles and thermo-responsive polyethylene glycol (PEG). Hilt and co-workers [61] published the procedure for the preparation of magnetic hydrogel composites based on NIPAAm monomer, PEG 400 dimethacrylate (PEG400DMA) as cross-linker and Fe_3_O_4_ nanoparticles (5 wt %) of 20–30 nm in diameter. When a disc of the hydrogel composite was subjected to an AMF (2.98 kA/m, 297 kHz) the temperature of the center of the disc increased from an initial temperature of 22 °C to *ca*. 55 °C within the first minute (Figure 11A). Hydrogels with no particles showed minimal resistive heating, while an increment in the particle loading increased the maximum temperature achieved under the magnetic field (Figure 11B). The authors also used pyrocatechol violet dye as a model drug to demonstrate its controlled release from the composite subjected to an AMF.

**Figure 12:**
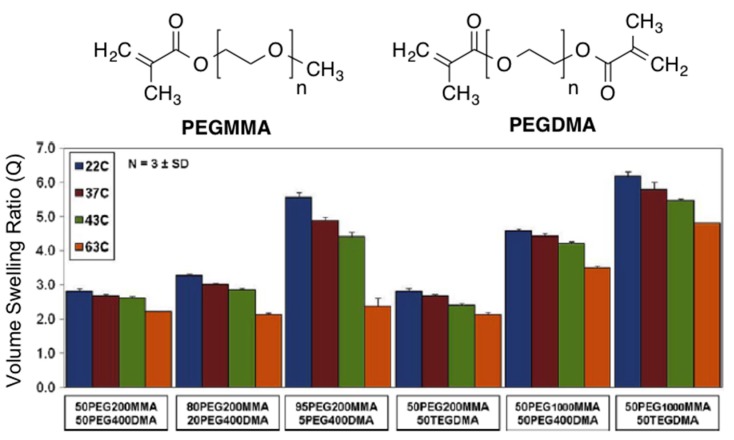
Structures of PEGMMA and PEGDMA (*top*) and swelling analysis results for all PEG hydrogel nanocomposites at 22, 37, 43, and 63 °C showing the volume swelling ratio (Q) for each system. Adapted with permission from reference [62]. Copyright ^©^ 2010 Elsevier.

The same group [62] published later the results obtained with a series of potentially biocompatible magnetic hydrogels prepared using Fe_3_O_4_ nanoparticles (Ø = 20–30 nm; 0.2% PVP-coated) and a polymer fabricated via free radical polymerization with various PEG methyl ether methacrylate (PEGMMA, macromer) and dimethacrylate (PEGDMA, cross-linking). TGA analyses demonstrated that the iron oxide loading was in the range 5.48 ± 0.65 wt % for all hydrogel nanocomposites, which was close to the initial loaded amount. Greater ethylene glycol (EG) amount and lower cross-linking density of the nanocomposites resulted in higher volume swelling ratios (Figure 12). In general, the swelling ratios decreased as they reached their approximate LCST and were slightly higher than those without nanoparticles probably due to less effective cross-linking during polymerization in the presence of the particles.

Both the entrapped iron oxide nanoparticles and hydrogel nanocomposites exhibited high viability using NIH 3T3 murine fibroblasts for cytotoxic tests with polystyrene controls, indicating potential biocompatibility. As in previous cases, when the hydrogels were heated in an AMF (*i.e.*, 5 min at 297 kHz and 25 kA/m), the heating response was shown to be dependent on both iron oxide loading in the gels and *H*. As the amount of iron oxide per volume in the gels increased, the final maximum temperature of the systems increased (Table 2). The hydrogel nanocomposites reached the maximum temperature after 3 min, whereas control hydrogels without magnetic particles exhibited minimal heating.

**Table 2:** Composition of hydrogel nanocomposites, murine fibroblast cell viability (%) for iron oxide nanoparticles and hydrogel nanocomposites at 24 h, final temperature values (°C) for the gels exposed to an AMF at 25 kA/m, calculated iron oxide mass for the heated gels (mg/cm^3^) and AMF strengths (kA/m) needed to achieve hyperthermia and thermo-ablative temperatures [62].

Macromer Feed	Cross-Linker Feed	Cell Viability	Final Temp.	Fe_3_O_4_ Mass/Gel	Hyperthermia AMF Strength	Thermo-ablative AMF Strength
50 mol % PEG200MMA	50 mol % PEG400DMA	95.7 ± 1.4	60.7 ± 0.7	1.58	17.3	25.3
80 mol % PEG200MMA	20 mol % PEG400DMA	97.3 ± 0.9	59.5 ± 1.1	1.92	17.4	25.9
95 mol % PEG200MMA	5 mol % PEG400DMA	97.9 ± 0.5	65.7 ± 1.7	2.74	16.5	24.2
50 mol % PEG200MMA	50 mol % TEGDMA	96.0 ± 2.0	66.1 ± 0.7	5.32	14.7	22.0
50 mol % PEG1000MMA	50 mol % PEG400DMA	97.0 ± 2.5	73.8 ± 0.8	7.24	14.3	23.0
50 mol % PEG1000MMA	50 mol % TEGDMA	96.9 ± 1.0	79.6 ± 1.3	7.93	12.7	17.4
1000 μg/mL Fe_3_O_4_		98.0 ± 0.2				
Control		97.9 ± 0.6				

**Figure 13:**
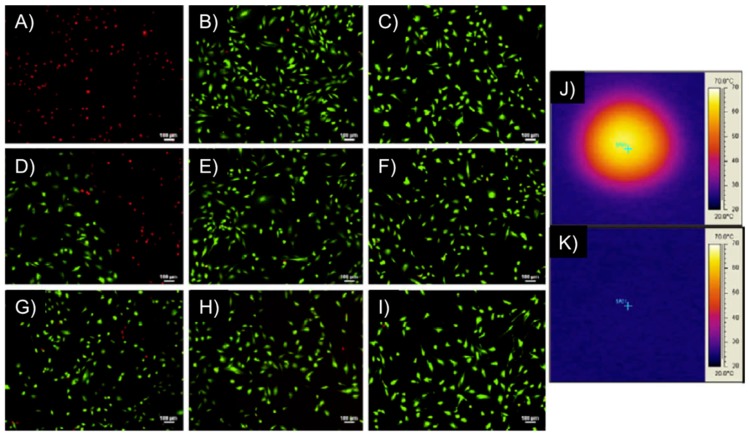
M059K glioblastoma multiform/hydrogel heating results. Images (**A**–**I**) represent fluorescent microscopy images after Live/Dead assay of M059K cells, where (**A**,**B**) are at the center of the Petri dish; (**D**–**F**) are at the interface between live and dead cells and (**G**–**I**) are at the outer edge, unaffected by heat. The first column of images are for cells exposed to a DM gel (50 mol % PEG200MMA, 50 mol % TEGDMA) at 297 kHz and 25 kA/m for 5 min, the middle column are of cells exposed to an AMF only at 297 kHz and 25 kA/m for 5 min and the right column is of cells not exposed to gels or an AMF; Images (**J**,**K**) represent IR images after the cells had been heated with the gel for 5 min (**J**) and exposed to an AMF for 5 min (**K**). Adapted with permission from reference [62]. Copyright ^©^ 2010 Elsevier.

Remarkably, the above-described PEGMMA/PEGDMA magnetic hydrogel nanocomposites were capable of heating to both hyperthermia (41–44 °C) and thermo-ablative temperatures (61–64 °C) depending on the strength of the AMF. The systems showed the ability to selectively kill M059K glioblastoma cells (*in vitro*) at the thermo-ablative temperature (63 °C) without the magnetic field causing harm (Figure 13). Although this study constituted the first one involving cell studies with such magnetic gels, experiments *in vivo* and demonstration of biocompatibility of the nanocomposites (before and after heating), potential nanoparticle release during implantation and evaluation of the overall effect of heating on the surrounding living tissues were not done.

**Figure 14:**
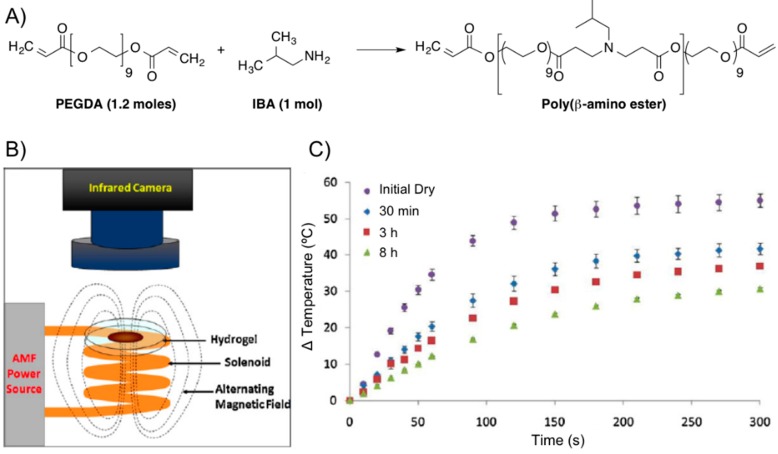
(**A**) Synthesis of poly(β-amino ester) from PEGDA and IBA; (**B**) scheme of AMF setup; and (**C**) thermal analysis of 0:100 2EG-IBA magnetic hydrogel nanocomposite with time upon exposure to an AMF of 17.4 kA/m and 294 kHz for 5 min (*n* = 3 ± SD). Adapted with permission from reference [64]. Copyright ^©^ 2012 Elsevier.

The same group [63] also prepared a poly(β-amino ester) (PBAE) biodegradable hydrogel composed of PEG (*n* = 400) diacrylate (PEG400DA) or DEGDA with isobutylamine (IBA). The combination with Fe_3_O_4_ nanoparticles (Ø = 20–30 nm, 0.2% PVP-coated) provided composites that were proved potentially useful for combined hyperthermia therapy and drug delivery in the synergistic treatment of cancer [58,59] enabling remote heating when an AMF was applied (Figure 14). Specifically, the authors performed first a solvent-free synthesis of two macromers via Michael addition reactions of IBA with excess PEGDA. For one macromer, a 1:1.2 molar ratio of IBA to DEGDA (with 2 ethylene glycol units, *n* = 2) was reacted for 48 h at 85 °C with mixing (thereby denoted as 2EG–IBA). The second macromer was composed of a 1:1.2 molar ratio of IBA to PEG400DA (9 EG units, *n* = 9) reacted for 15 h at 85 °C (denoted 9EG–IBA). The magnetic hydrogel nanocomposites were then fabricated by free-radical polymerization with various ratios of the two macromers. The resulting systems had 0, 25, 50, 75, and 100 wt % 2EG-IBA to 9EG-IBA macromer (100:0, 75:25, 50:50, 25:75, and 0:100 2EG-IBA). 5 wt % Fe_3_O_4_ nanoparticles and 5 mg of paclitaxel (PTX, Taxol^®^) per mg of macromer were also incorporated into the formulation. The obtained gel composites were exposed to an electromagnetic field for 5 min at 294 kHz and 17.4 kA/m to induce heating within the systems for the following conditions: dry hydrogels and hydrogels after degradation at various time points. The hydrogels were able to heat for all conditions and the maximum change in temperature reached was suitable for hyperthermia and decreased as the gels degraded (Figure 14C).

**Figure 15:**
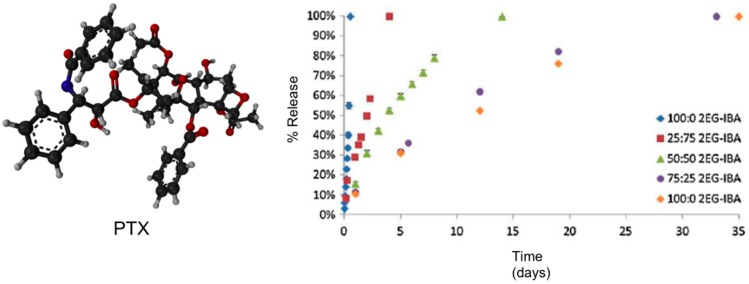
Ball-and-stick molecular model of PTX (paclitaxel, Taxol^®^) and its release from hydrogel nanocomposites over time via HPLC. *n* = 3 ± SD. Adapted with permission from reference [64]. Copyright ^©^ 2013 Taylor & Francis.

Kinetic studies showed that both the degradation and PTX release profiles (near-zero-order rate for all systems) could be tailored based on the type of macromer used with the more hydrophilic hydrogel systems (*i.e.*, 0:100 2EG-IBA) degrading in 11 h *versus* over nearly seven weeks for the more hydrophobic system (100:0 2EG-IBA) (Figure 15). This data showed the ability to tailor the degradation and swelling profiles of the composites by adjusting the macromer content. PTX exhibited non-Fickian release [64] and it was controlled by the degradation of the hydrogels through bulk degradation upon exposition to an AMF. Importantly, the authors also evaluated the cytotoxicity of the degradation products against NIH 3T3 murine fibroblasts exhibiting cell viabilities from 53% to 92% depending on the type of hydrogel and concentration of degradation products. This study was also expanded to other cell lines including MDA MB 321 (breast carcinoma) and A549 (lung adenocarcinoma). *In vitro* cell studies demonstrated that combined PTX and hyperthermia treatment increased the efficacy of PTX for A549 lung carcinoma cells.

**Figure 16:**
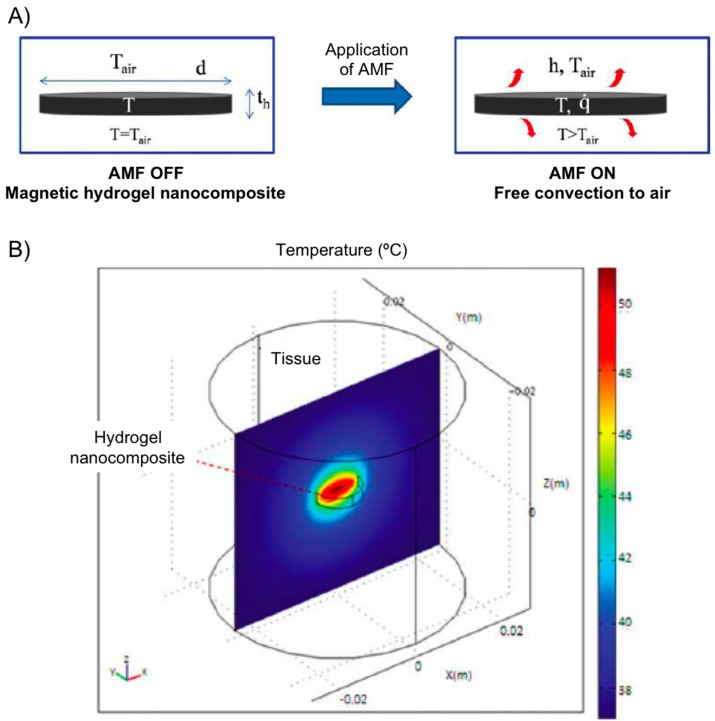
(**A**) Heat generation of a magnetic hydrogel nanocomposite disc subjected to AMF is by magnetic nanoparticles and loss is by convection to surrounding air; and (**B**) temperature profile at steady state for hydrogel disc (radius 5 mm, thickness 2 mm, particles 5 wt %, AMF 25 kA/m) and surrounding tissue in *x*–*z* plane along *y* = 0. Adapted with permission from reference [65]. Copyright ^©^ 2011 American Institute of Chemical Engineers.

More recently, using PEG hydrogel nanocomposites containing iron oxide loading between 0 and 5 wt %, Hilt’s group [65] developed a heat transfer mathematical model for predicting temperature profiles of a hydrogel disc heated with an AMF in air environment (Figure 16). The hydrogel disc was covered in Saran wrap and suspended on top of the solenoid. In this set up, the temperature of the nanocomposite at any time depends on the rate of heat generation and the rate of heat loss to surroundings by convection. Experimental data were collected and AMF amplitudes of 14.8, 19.5, and 25 kA/m at 293 kHz. The model successfully predicted temperatures of a PEG hydrogel system with different swelling characteristics. For *in vivo* predictions, temperature profiles of a hydrogel disc and surrounding tissue were simulated using modeling software COMSOL3.4. Although *in vivo* conditions resulted in lower hydrogel temperatures, it should be emphasized that heating profiles can be influenced by hydrogel geometry, particle loadings, and AMF amplitude allowing for further optimization.

**Figure 17:**
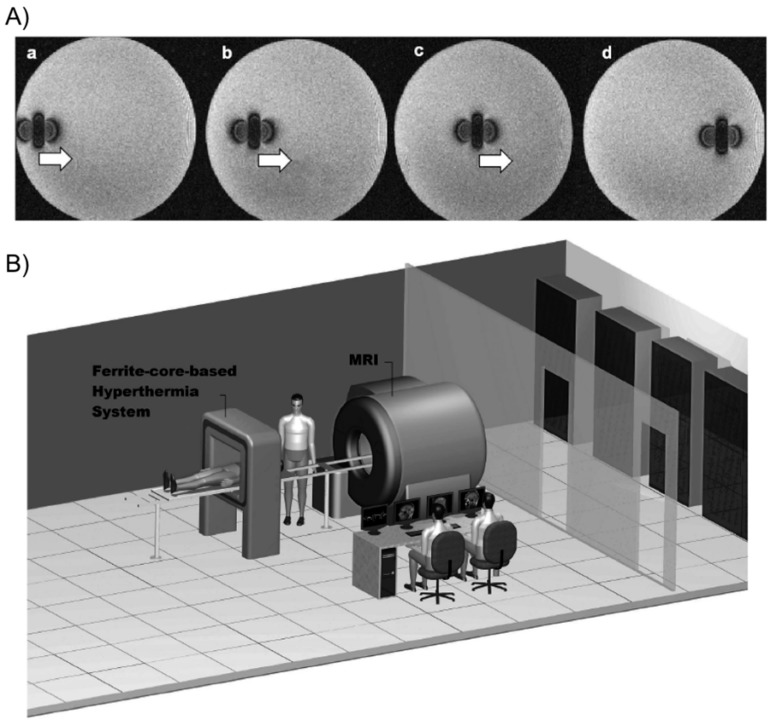
(**A**) MRI images of the microrobot at four different positions. The artifact caused by the microrobot approximates the magnetic dipole field that it induces, making it possible to track its position; and (**B**) overview of the magnetic resonance tracking and guiding platform integrated with the magnetic hyperthermia system. The patient can easily be transferred from one system to another for a complete delivery procedure. Adapted with permission from reference [66]. Copyright ^©^ 2011 Taylor & Francis.

Tabatabaei and co-workers [66] described the preparation of microrobots containing ferromagnetic or superparamagnetic nanoparticles encapsulated in thermo-sensitive PNIPAm hydrogels. For potential targeted drug delivery, these composites were able to shrink (25% volume reduction) in response to temperature when exposed to an AMF of 4 kA/m at 160 kHz. In addition, they could be propelled in the vascular network while being tracked for navigation control purposes using magnetic gradients of 400 mT/m generated by a clinical magnetic resonance imaging (MRI) scanner (Figure 17).

Korotych’s group [67] demonstrated that the *in situ* synthesis of Fe_3_O_4_ particles in nanoreactors of thermo-sensitive co-polymeric hydrogels (*i.e.*, made of *N*-isopropylacrylamide (NIPAAm), acrylamide (AAm) and *N*,*N′*-methylenebisacrylamide (MBA) as cross-linker) permitted their stabilization and prevention of nanoparticle aggregation and allowed for obtaining magnetic particles with an average size of about 20 nm (0.2% of MBA). Co-polymer ferrogels having 95% of NIPAAm and 5% of AAm with 0.2% MBA content showed to possess the best properties for biomedical application. Ferrogels with such composition were characterized by an abrupt phase transition at about 42 °C and by high magneto-sensitivity that grew with the increase of incorporated magnetite concentration, along with the decrease of the hydrogel cross-linking density. The materials were characterized by critical iron concentrations (higher at higher AAm content) at which the networks collapsed. Moreover, ferrogels containing significant amounts of magnetite (up to 30%–50% depending on the cell lines) did not show cytotoxicity.

**Figure 18:**
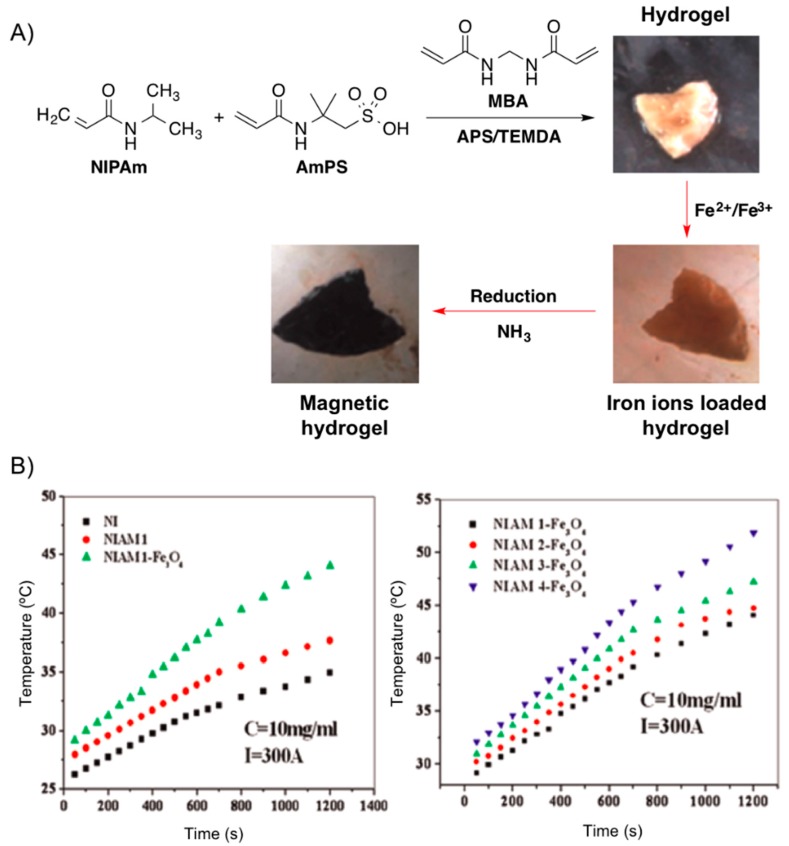
(**A**) Synthesis of Fe_3_O_4_ nanoparticles throughout NIPAM-AmPS hydrogel networks. (**B**) *Left*: Heating curves of pure NI (system without AmPS; black squares), NIAM (hydrogel without nanoparticles; red circles) and NIAM-Fe_3_O_4_ (composite hydrogel; green triangles). *Right*: Heating curves for different hydrogel magnetic nanocomposite at fixed apparent current (*I* = 300 A). Adapted with permission from reference [68]. Copyright ^©^ 2015 Elsevier.

Very recently, Reddy and co-workers [68] reported the development of metal absorber, thermo- and magnetic-responsive hydrogel networks by polymerizing NIPAm in the presence of AmPS using MBA as cross-linker and the mixture ammonium persulphate (AmPS)/tetramethyl ethylenediamine (TMEDA) as redox initiating system. The magnetic nanoparticles were generated throughout the hydrogel network using *in situ* method by incorporating Fe^2+^ and Fe^3+^ ions and subsequent treatment with ammonia (Figure 16). The increase of AmPS content in the hydrogel networks resulted in higher amounts of Fe_3_O_4_ formed in the hydrogels, which was accompanied by an increment of the heating effect necessary for hyperthermia applications (Figure 18).

## 3. Conclusions and Future Perspectives

Some technological challenges of hyperthermia cancer therapy involve the necessity of achieving localized and uniform temperature in the tumor, and the ability to precisely monitor the temperatures of both the tumor and the surrounding tissue. In this sense, the incorporation of magnetic micro/nanoparticles into biocompatible and thermo-responsive polymer hydrogels represents a promising approach for magnetic hyperthermia cancer therapy. Mainly due to the hysteresis loss from the magnetic particles subjected to an external magnetic field at fixed frequency and amplitude, the temperature of the system increases and once the temperature crosses the lower critical solution temperature, thermo-responsive hydrogels undergo large contraction. Such collapse transition can be accompanied by the controlled release of anti-cancer drug molecules that have been previously entrapped in the gel networks. Moreover, hydrogels can provide increased biocompatibility over exposed, uncoated nanoparticles due to an encapsulation effect within the composite matrix. Numerous studies in this area have demonstrated that the maximum temperature achieved by the composites could be easily adjusted on-demand based on the type of polymer gel network, the properties and concentration of the magnetic particles, the method of incorporation of the magnetic particles, and/or the frequency and intensity of the magnetic field.

The tailorability of magnetic gel composites makes them solid candidates as soft-actuators for the synergistic treatment of cancer involving both triggered drug release and thermal therapy via magnetic hyperthermia. However, there are still a number of challenges that need to be addressed in future research before expanding the clinical applications. Among them, some of the most important aspects are: (1) the necessity of more *in vivo* testing including the long-term fate of embedded magnetic particles; (2) the study of the physiological parameters (e.g., blood viscosity and velocity, tissue conductivity) affecting the functionality of implanted composites in the human vascular network (e.g., blood perfusion may lead to lower temperatures than expected) or the reconstitution of the functional gels after injection; (3) the quantitative assessment of the number of particles per cell required for a lethal effect in specific tumors; (4) the possible necessity to surgically remove the implant after its useful life; (5) the use of new formulations to avoid overheating effects and maximize the available heating efficiency while minimizing the amount of magnetic particles; (6) the development of pharmacological targeting particles (e.g., antibody-nanoparticle conjugates) and hydrogels to improve specific molecular recognition and intratumoral accumulation; and (7) the development of more general predicting models will be helpful for the rational design of new polymers to be exposed to a variety of heat transfer environments.

Despite all these challenges, the development of magnetic gel composites with good response properties, even under other activation methods [69,70,71,72], and controllability will greatly promote the advance of biomedical engineering and cancer treatment.
